# Supplementary material for: Comparative Performances of Beneficial Microorganisms on the Induction of Durum Wheat Tolerance to Fusarium Head Blight
Source: Microorganisms. 2021 Nov 23;9(12):2410. doi: 10.3390/microorganisms9122410 (PMC8705052; doi:10.3390/microorganisms9122410)
Supplement: Supplementary file 1 [file microorganisms-09-02410-s001.zip › microorganisms-1418577-supplementary.pdf]

Table S1. ANOVA Effect of treatments on FHB severity in durum wheat evaluated in three-year-study.

variables	**DF**	**Mean squares**
Treatments (T)	5	2143.7***
Year (Y)	2	1335.0***
T X Y	10	90.7***

The mean squares are shown, and the symbols indicate statistical significance (.,p≤ 0. 1; ***, p≤0.001).

Table S2. ANOVA Effect of treatments on yield components of durum wheat evaluated in three-year-study.

**traits**		**Grain yield (Mg/ha)**	**Thousand Kernels weigh (TKW) (g)**
variables	**DF**	**Mean squares**	
Treatments (T)	5	14.885***	22.2***
Year (Y)	2	14.731***	666.5***
T X Y	10	2.565***	8.3***

The mean squares are shown, and the symbols indicate statistical significance (·,*p*≤ 0. 1; ***, *p*≤0.001).

Table S3. ANOVA Effect of the biostimulants on the physiological traits and carbon and nitrogen isotope discrimination in the 2018 cropping season.

	Mean squares							
**Traits**	**GY (Mg/ha)**		**TKW (g)**	**FHB (%)**	**NDVI**	**SPAD**	**CT (°C)**	**SC (mmol.m^{-2}·s^{-1})**
Treatments (T) (Df=5)	7.511***		18.379**	728.6***	0.009430***	24.580***	6.796***	2229.9*

**Traits**	**C_{leaf}(%, g DW)**	**$\delta^{13}C_{leaf}$ (‰)**	**N_{leaf} (%, g DW)**	**$\delta^{15}N_{leaf}$ (‰)**	**C_{grain} (%, g DW)**	**$\delta^{13}C_{grain}$ (‰)**	**N_{grain} (%, g DW)**	**$\delta^{15}N_{grain}$ (‰)**
Treatments (T) (Df=5)	4.910***	1.0093***	1.5433***	2.5780***	3.809***	0.11692***	0.06099***	1.4316***

The mean squares are shown, and the symbols indicate statistical significance (*, p≤0.5;**, p≤0.01; ***, p≤0.001).
